# Analysis of Rates of Brain Metastases and Association With Breast Cancer Subtypes in Ontario, Canada

**DOI:** 10.1001/jamanetworkopen.2022.25424

**Published:** 2022-08-12

**Authors:** Xin Ye Wang, Michael N. Rosen, Rania Chehade, Arjun Sahgal, Sunit Das, Ellen Warner, Refik Saskin, Bo Zhang, Hany Soliman, Kelvin K. W. Chan, Katarzyna J. Jerzak

**Affiliations:** 1 Institute of Medical Sciences, Faculty of Medicine, University of Toronto, Toronto, Ontario, Canada; 2 Faculty of Medicine, University of Ottawa, Ottawa, Canada; 3 Division of Medical Oncology, Sunnybrook Odette Cancer Centre, University of Toronto, Toronto, Ontario, Canada; 4 Department of Radiation Oncology, Sunnybrook Odette Cancer Centre, University of Toronto, Toronto, Ontario, Canada; 5 Division of Neurosurgery, St Michael’s Hospital, University of Toronto, Toronto, Canada; 6 Institute of Health Policy, Management and Evaluation, University of Toronto, Toronto, Ontario, Canada; 7 Institute of Clinical Evaluative Sciences, Sunnybrook Health Sciences Centre, Sunnybrook Research Institute, Toronto, Ontario, Canada

## Abstract

**Importance:**

Approximately 1 in 7 patients with metastatic breast cancer (MBC) will receive radiotherapy for brain metastases (BRM). Significant differences in cumulative incidence of BRM by breast cancer subtype may inform future BRM screening protocols.

**Objective:**

To describe cumulative incidence of BRM among patients with de novo MBC.

**Design, Setting, and Participants:**

In this population-based cohort study, population health administrative databases in Ontario, Canada, held at the ICES were used to identify patients diagnosed with de novo MBC between 2009 and 2018. Given that a code for BRM does not exist within ICES, we analyzed the incidence of radiotherapy for BRM. The median (IQR) follow-up was 19.3 (6.2-39.5) months. A total of 100 747 patients with a new diagnosis of breast cancer between January 2009 and December 2018 were identified. Of these patients, 17 955 were excluded because they had previous or subsequent malignant neoplasms, 583 were excluded because they were younger than 18 years, 974 were excluded because there was an invalid Ontario Health Insurance Plan number or a date of death on or before the index date. Among 81 235 remaining patients, 3916 were identified as having de novo MBC.

**Exposures:**

Treatment with radiotherapy for breast cancer BRM.

**Main Outcomes and Measures:**

Cumulative incidence of radiotherapy for BRM accounting for the competing risk of death, and time from MBC diagnosis to brain radiotherapy. Kaplan-Meier analyses were performed for time-to-event end points. Logistic regression was used to account for confounding variables.

**Results:**

Among 3916 patients with MBC, 1215 (31.0%) had HR-positive/*ERBB2* (formerly *HER2*)-negative cancer, 310 (7.9%) had *ERBB2*-positive/HR-positive cancer, 200 (5.1%) had *ERBB2*-positive/HR-negative cancer, 258 (6.6%) had TNBC, and the remaining 1933 patients (49.4%) had an unknown breast cancer subtype. The median (IQR) age at diagnosis was 63 (52-75). A total of 549 (14.0%) underwent stereotactic radiosurgery or whole brain radiotherapy for breast cancer BRM. Cumulative incidence of BRM was higher among patients with *ERBB2*-positive/HR-negative breast cancer (34.7%), *ERBB2*-positive/HR-positive breast cancer (28.1%), and triple-negative breast cancer (21.9%) compared to those with HR-positive/*ERBB2*-negative breast cancer (12.1%). The median (IQR) time from MBC diagnosis to brain radiotherapy ranged from 7.5 (2.3-17.4) months for patients with TNBC to 19.8 (12.2-35.1) months for those with *ERBB2*-positive/HR-positive breast cancer.

**Conclusions and Relevance:**

Incidence and time to development of BRM vary significantly by breast cancer subtype. A better understanding of the biology of intracranial metastatic disease may help inform potential screening programs or preventative interventions.

## Key Points

**Question:**

What is the cumulative risk of brain metastases (BRM) requiring radiotherapy among patients with metastatic breast cancer (MBC)?

**Findings:**

In this population-based cohort of 3916 patients with de novo MBC, 14.0% required radiotherapy for BRM; this proportion was highest among patients with *ERBB2* (formerly *HER2*)–positive/hormone receptor (HR)–negative (34.7%), *ERBB2-*positive/HR-positive (28.1%), and triple-negative breast cancer (TNBC; 21.9%). The median time from MBC diagnosis to brain radiotherapy varied significantly by subtype.

**Meaning:**

These findings suggest that a high proportion of patients with metastatic *ERBB2*-positive breast cancer and TNBC will require radiotherapy for BRM; varying incidence and time to development of BRM by breast cancer subtype could inform future BRM screening programs.

## Teaser

This cohort study describes clinical cumulative incidence of brain metastases among patients with de novo metastatic breast cancer using population health administrative databases in Ontario, Canada.

## Introduction

Brain metastases (BRM) are a major cause of morbidity and mortality in women with breast cancer.^1,2,3,4,5^ Women with metastatic *ERBB2* (formerly *HER2*)–positive and triple-negative breast cancer (TNBC) have a particularly high propensity to develop intracranial disease; in fact, over one-third of these women develop BRM during their lifetime.^6,7,8,9,10^ Although some systemic therapies have demonstrated intracranial activity, the mainstay of therapy for patients with breast cancer BRM remains local therapy, with radiation in the form of whole brain radiotherapy (WBRT) and/or stereotactic radiosurgery (SRS), as well as surgery in select cases.

Despite the large burden of symptomatic BRM on patients and the health care system, current guidelines do not recommend the use of neuroimaging to routinely screen for BRM in women with early-stage or metastatic breast cancer (MBC).^11,12,13,14^ We hypothesize that early detection of BRM in patients with MBC via a magnetic resonance imaging (MRI)-based screening program may allow for early intervention and improved outcomes. However, appropriate data to inform clinical trials or policies of screening MBC patients for BRM are lacking.

Most of the relevant data in the literature is retrospective in nature without an inception cohort, resulting in inherent risks of bias. Further, in the few prospective studies the incidence of actionable BRM (ie, proportion of patients who are sufficiently fit to receive treatment for BRM) and the timelines from MBC diagnosis to treatment for BRM are unclear.

We used Ontario-wide health administrative data to identify the cumulative probability of receiving radiotherapy for BRM among women with de novo MBC as well as the time to development of BRM from the time of MBC diagnosis, grouped according to hormone receptor (HR) and *ERBB2* status. Our main research question related to the cumulative incidence of receiving radiotherapy for BRM among patients with triple-negative or *ERBB2*-positive disease, who are at highest risk of developing BRM. We hypothesized that cumulative risk of BRM and corresponding survival rates may inform the timing and duration of future BRM screening or prevention programs for patients who are at high risk of developing intracranial metastases.

## Methods

This retrospective, observational population-based cohort study was approved by the research ethics board at Sunnybrook Research Institute, and informed consent was waived because ICES is a prescribed entity under Ontario’s Personal Health Information Protection Act. The Strengthening the Reporting of Observational Studies in Epidemiology (STROBE) reporting guideline was followed.

We assessed the cumulative incidence of first radiotherapy for intracranial metastatic disease among patients diagnosed with de novo MBC between January 2009 and December 2018, stratified by subtype. Time to treatment with radiotherapy (WBRT or SRS for intracranial metastatic disease) and cumulative risk of death were also captured. Patients with de novo MBC were identified because metastatic relapse after diagnosis of early breast cancer is not captured in population health administrative databases in Ontario, Canada, which are held at ICES. ICES is an independent, nonprofit research institute funded by an annual grant from the Ontario Ministry of Health and Long-Term Care (MOHLTC). Unfortunately, we were not able to distinguish between parenchymal BRM vs leptomeningeal disease in this study.

### Measures and Data Sources

Variables of interest for data collection included breast cancer subtype (hormone receptor [HR] positive/*ERBB2 *negative, *ERBB2 *positive [either HR negative or HR positive], triple-negative breast cancer [TNBC] and unknown subtype), patient age, type of brain radiotherapy (SRS vs WBRT), residence setting (urban vs rural),^15^ VW Elixhauser weighted comorbidity index,^16^ income status,^17^ and index year at diagnosis. American Joint Committee on Cancer (AJCC) disease stage at diagnosis was reported according to the Collaborative Staging Methodology.^18^ Rates of radiation therapy were calculated using radiation exposure data captured in the Ontario Health Insurance Plan (OHIP), National Ambulatory Care Reporting System (NACRS), or Activity Level Reporting (ALR) databases between diagnosis and study end (eTable 1 in the Supplement).

Cumulative incidence of radiotherapy for BRM and the time-to-radiotherapy for BRM were analyzed by breast cancer subtype: HR-positive/*ERBB2-*negative, *ERBB2-*positive (either HR negative or HR positive), TNBC and unknown subtype. The unknown group includes patients whose HR or *ERBB2* status were not available or incomplete. To identify factors associated with the development of BRM, characteristics of patients who did vs did not develop BRM were compared. Finally, the median survival times from first radiotherapy for BRM to death were calculated.

### Statistical Analyses

The primary outcomes were cumulative incidence of radiotherapy for BRM at 1, 2, 3, 5 and 9 years, as well as time-to-radiotherapy for the treatment of BRM, defined as time from diagnosis of MBC to the time of radiotherapy for BRM. Overall survival (OS), defined as time from radiotherapy for BRM to the time of death due to any cause was also assessed as a secondary outcome. Data were censored if patients were alive on the same therapy at last available follow-up with the last cut-off date being March 31, 2019. Categorical variables are presented as a percentage of nonmissing cases and continuous variables are reported as mean (SD) or median (IQR). Differences between patients receiving SRS vs WBRT were compared using Wilcoxon tests for continuous variables and χ^2^ tests for categorical variables.

Cumulative incidence of radiotherapy for BRM from the diagnosis of de novo MBC was calculated using the Cumulative Incidence Function (CIF), accounting for the risk of death using a competing risk analysis; corresponding percentage incidence at 1-, 2-, 3-, 5- and 9-year time points were indicated in tabular format. Characteristics of patients who were and were not treated with radiotherapy for breast cancer BRM were compared using a Fine-Gray model. Kaplan-Meier analyses were performed for the time to event end point (OS) and compared using the log-rank test. Data were analyzed using SAS from January 2021 to June 2022. A 2-sided *P* < .05 was considered statistically significant, with *P* values for difference in medians estimated using a Kruskal-Wallis test and *P* values for categorical values derived from a χ^2^ test.

## Results

Baseline characteristics of all patients diagnosed with de novo MBC are shown in Table 1. A total of 100 747 patients with a diagnosis of breast cancer were identified between January 2009 and December 2018. Patients were excluded if they had previous or subsequent malignant neoplasms (17 955 patients), were younger than 18 years (583 patients), or had an invalid OHIP or a date of death on or before the index date, which was likely due to erroneous data entry (974 patients). Among the 81 235 remaining patients, 3916 were identified as having de novo metastatic (stage IV) breast cancer (eTable 2 in the Supplement); among them, 549 (14.0%) patients underwent SRS or WBRT for breast cancer BRM. The baseline characteristics of patients who were treated with SRS or WBRT for breast cancer BRM are described in eTable 3 in the Supplement. The median (IQR) follow-up from time of MBC diagnosis among patients with BRM was 25.1 (12.4-40.6) months.

**Table 1.** Baseline Characteristics of Patients With Metastatic Breast Cancer by Breast Cancer Subtype (N = 3916)

Variable	Patients, No. (%)						*P* value
	TNBC (n = 258)	*ERBB2*+/HR+ (n = 310)	*ERBB2*+/HR− (n = 200)	HR+/*ERBB2*− (n = 1215)	Unknown (n = 1933)	All (N = 3916)	
Age at treatment, y							
Median (IQR)	65 (51-76)	59 (49-71)	57 (47-67)	63 (53-74)	64 (53-77)	63 (52-75)	<.001
≤60	106 (41.1)	162 (52.3)	115 (57.5)	527 (43.4)	804 (41.6)	1714 (43.8)	<.001
>60	152 (58.9)	148 (47.7)	85 (42.5)	688 (56.6)	1129 (58.4)	2202 (56.2)	
Location							
Urban	233 (90.3)	273 (88.1)	174 (87.0)	1104 (90.9)	1710 (88.5)	3494 (89.2)	.18
Rural	25 (9.7)	37 (11.9)	26 (13.0)	111 (9.1)	223 (11.5)	422 (10.8)	
Income, quintile							
1st	59 (22.9)	70 (22.6)	30 (15.0)	283 (23.3)	391 (20.2)	833 (21.3)	.03
2nd	59 (22.9)	73 (23.5)	49 (24.5)	290 (23.9)	427 (22.1)	898 (22.9)	
3rd	60 (23.3)	45 (14.5)	41 (20.5)	214 (17.6)	408 (21.1)	768 (19.6)	
4th	48 (18.6)	66 (21.3)	37 (18.5)	209 (17.2)	366 (18.9)	726 (18.5)	
5th	32 (12.4)	56 (18.1)	43 (21.5)	219 (18.0)	341 (17.6)	691 (17.6)	
Elixhauser comorbidity index score							
<0	36 (14.0)	46 (14.8)	21 (10.5)	161 (13.3)	280 (14.5)	544 (13.9)	.003
0	101 (39.1)	124 (40.0)	82 (41.0)	469 (38.6)	766 (39.6)	1542 (39.4)	
1-4	70 (27.1)	103 (33.2)	61 (30.5)	318 (26.2)	460 (23.8)	1012 (25.8)	
≥5	51 (19.8)	37 (11.9)	36 (18.0)	267 (22.0)	427 (22.1)	818 (20.9)	
Index year at diagnosis							
2009-2010	0	0	0	1-5	747-751	752 (19.2)	<.001
2011-2012	46 (17.8)	50 (16.1)	40 (20.0)	177-181	435-439	752 (19.2)	
2013-2014	78 (30.2)	96 (31.0)	55 (27.5)	400 (32.9)	158 (8.2)	787 (20.1)	
2015-2016	92 (35.7)	113 (36.5)	71 (35.5)	412 (33.9)	127 (6.6)	815 (20.8)	
2017-2018	42 (16.3)	51 (16.5)	34 (17.0)	221 (18.2)	462 (23.9)	810 (20.7)	

Abbreviations: HR, hormone receptor; TNBC, triple-negative breast cancer.

### Baseline Patient Characteristics

In the overall patient population, 1215 (31.0%) had HR-positive/*ERBB2-*negative cancer, 310 (7.9%) had *ERBB2-*positive/HR-positive cancer, 200 (5.1%) had *ERBB2-*positive/HR-negative cancer, 258 (6.6%) had TNBC, and the remaining 1933 patients (49.4%) had an unknown breast cancer subtype. The median (IQR) age at diagnosis was 63 (52-75); 3494 patients (89.2%) lived in an urban residence and 2086 patients (53%) had a weighted Elixhauser comorbidity index of 0 or less.

Patients who received radiotherapy for BRM were more likely to have TNBC or *ERBB2-*positive cancer than HR-positive/*ERBB2-*negative cancer (*P* < .001) and were younger than those who did not receive radiotherapy for BRM (*P* < .001). The residence of patients (urban vs rural) did not differ between the 2 groups.

The median (IQR) survival time from diagnosis of MBC was 19.3 (6.2-39.5) months in the overall cohort and 26.2 (14.5-42.8) months for HR-positive/*ERBB2*-negative cancer, 27.8 (13.3-44.3) months for *ERBB2*-positive/HR-positive cancer, 22.9 (8.1-42.6) months for *ERBB2*-positive/HR-negative cancer, 8.8 (3.5-17.5) months for TNBC, and 13.2 (4.2-37.4) months for unknown subtypes.

### Cumulative Incidence of Brain Metastases Receiving Radiotherapy

The cumulative incidence of BRM (identified by treatment with radiotherapy to the brain) and cumulative incidence of death in the overall patient population is illustrated in the Figure and detailed in eTable 4 in the Supplement. Very few patients had BRM at the time of MBC diagnosis, as defined by radiotherapy to the brain within 4 weeks of being diagnosed with metastatic disease (≤2.5%).

**Figure.**
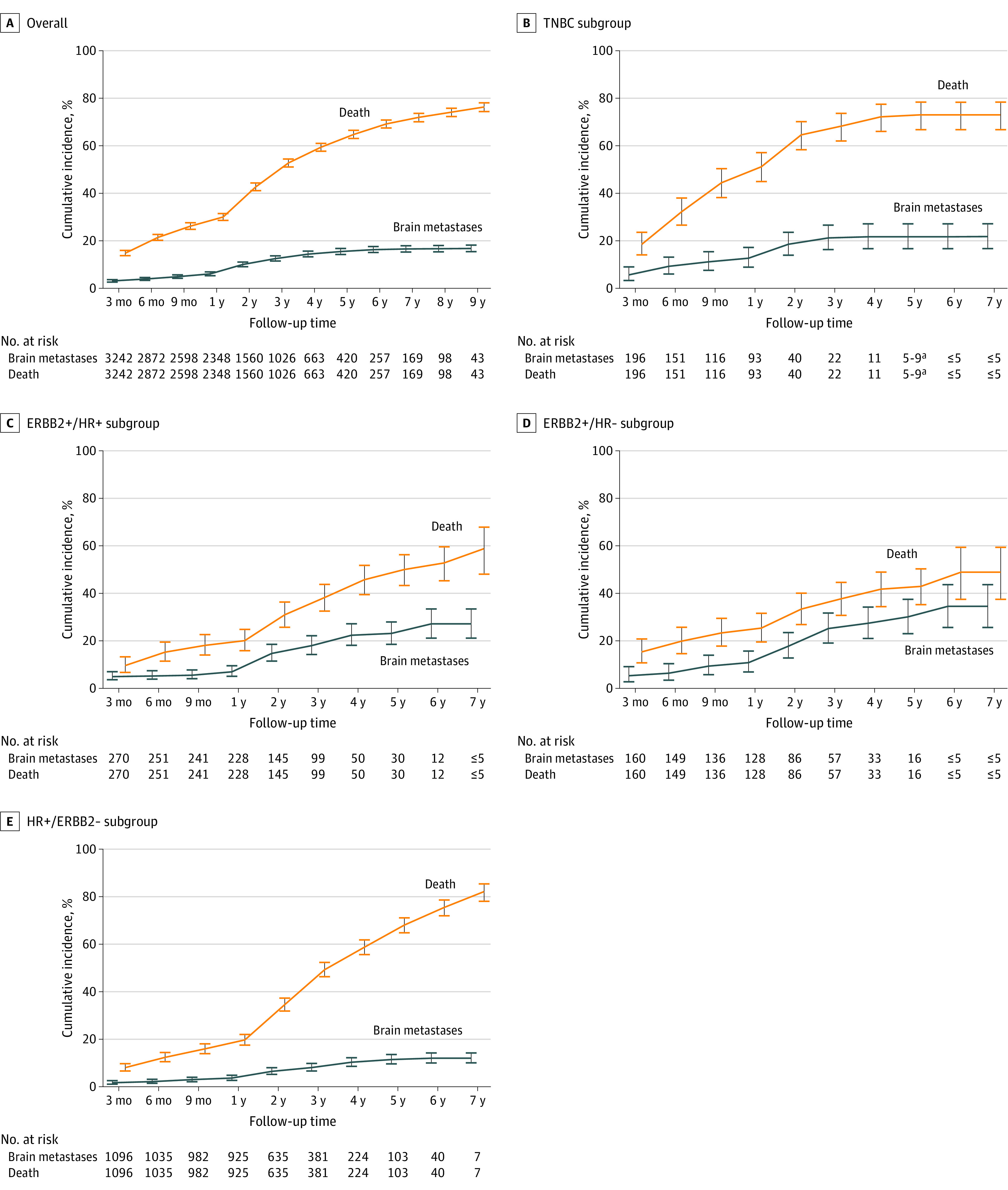
Cumulative Incidence of Brain Metastases and Cumulative Incidence of Death Among Patients With Metastatic Breast Cancer by Breast Cancer Subtype Cumulative incidence of brain metastases and survival among patients with metastatic breast cancer by breast cancer subtype. HR indicates hormone receptor; TNBC, triple-negative breast cancer.

The cumulative incidence of BRM at the 1-year time point for patients with HR-positive/*ERBB2-*negative cancer was 3.8% (95% CI, 2.8%-5.0%), *ERBB2-*positive/HR-positive cancer was 5.2% (95% CI, 3.1%-8.1%), *ERBB2-*positive/HR-negative cancer was 11.0% (95% CI, 7.1%-15.8%), and triple-negative MBC was 12.9% (95% CI, 9.1%-17.4%) (Figure). Corresponding cumulative incidence of BRM at the 3-year time point for patients with HR-positive/*ERBB2-*negative cancer was 8.2% (95% CI, 6.7%-9.9%), *ERBB2-*positive/HR-positive cancer was 17.7% (95% CI, 13.5%-22.4%), *ERBB2-*positive/HR-negative cancer was 25.3% (95% CI, 19.2%-31.8%), and triple-negative MBC was 21.4% (95% CI, 16.5%-26.8%) (Figure).

### Time to Development of Breast Cancer Brain Metastases

The median (IQR) time from MBC diagnosis to treatment for BRM with radiotherapy was 461 (137-900) days (ie, approximately 15 months) in the overall patient population. The median (IQR) time from diagnosis of MBC to first radiotherapy treatment for BRM was 16.8 (7.1-34.7) months in HR-positive/*ERBB2-*negative cancer, 19.8 (12.2-35.1) months in *ERBB2-*positive/HR-positive cancer, 15.0 (7.1-30.1) months in *ERBB2-*positive/HR-negative cancer, 7.5 (2.3-17.4) months in TNBC, and 14.1 (3.0-30.1) months in unknown subtypes (Table 2).

**Table 2.** Incidence Proportion, Time From Diagnosis to Radiotherapy to the Brain, and Median Survival of Patients With Metastatic Breast Cancer and Brain Metastases by Breast Cancer Subtype

Breast cancer subtype	Incidence proportion of BRM, WBRT and SRS among Women with MBC				Median (IQR), months				*P* value^a^
	No. (%)			*P* value^a^	Time from diagnosis of MBC to BRM	Survival time (from diagnosis of BRM to death)			
	Total BRM	WBRT	SRS			Total	WBRT	SRS	
TNBC	258 (20.9)	46 (17.8)	8 (3.1)	<.001	7.5 (2.3-17.4)	2.6 (1.3-6.7)	2.4 (1.1-6.7)	3.3 (2.5-6.1)	.57
*ERBB2*+/HR+	310 (20.6)	41 (13.2)	23 (7.4)	.02	19.8 (12.2-35.1)	8.7 (3.1-22.3)	7.1 (2.9-19.3)	12.2 (7.7-28.7)	.13
*ERBB2*+/HR−	200 (27.0)	40 (20.0)	14 (7.0)	<.001	15.0 (7.1-30.1)	9.4 (4.4-21.3)	7.4 (2.8-15.5)	19.5 (9.7-32.7)	.01
HR+/*ERBB2*−	1215 (10.1)	107 (8.8)	16 (1.3)	<.001	16.8 (7.1-34.7)	4.8 (1.7-12.9)	3.1 (1.3-12.5)	13.1 (7.3-23.9)	<.001
Unknown	1933 (13.1)	221 (11.4)	33 (1.7)	<.001	14.1 (3.0-30.1)	3.9 (1.4-10.4)	3.2 (1.3-9.4)	9.3 (4.1-14.5)	<.001
All subtypes	3916 (14.0)	455 (11.6)	94 (2.4)	<.001	15.4 (4.6-30.0)	5.1 (1.7-12.8)	3.7 (1.4-11.2)	11.4 (5.6-23.1)	<.001

Abbreviations: BRM, brain metastases; HR, hormone receptor; MBC, metastatic breast cancer; SRS, stereotactic radiosurgery; TNBC, triple-negative breast cancer; WBRT, whole brain radiotherapy.

^a^ *P* value compares WBRT and SRS groups.

Among all women with MBC (n = 3916), the TNBC (hazard ratio, 4.25; 95% CI, 3.05-5.84; *P* < .001) subtype was most strongly associated with a shorter time from diagnosis of metastatic disease to treatment of BRM with radiotherapy, taking into account the competing risk of death in a multivariable model that adjusted for the index year of diagnosis as well as other potential confounding variables **(**Table 3). A shorter time from MBC diagnosis to BRM treatment was associated with having *ERBB2-*positive/HR-positive subtype (hazard ratio, 1.94; 95% CI, 1.43-2.62; *P* < .001), *ERBB2-*positive/HR-negative subtype (hazard ratio, 2.81; 95% CI, 2.02-3.85; *P* < .001) and being age 60 years or younger (hazard ratio, 1.64; 95% CI, 1.38-1.96; *P* < .001).

**Table 3.** Multivariable Cox Model for Time (Years) From Diagnosis of Metastatic Breast Cancer to Brain Metastases (Events = 549; N = 3916) Using Competing-Risks Data

Variable	Hazard ratio (95% CI)	*P* value
Breast cancer subtype^a^		
TNBC vs HR+/*ERBB2*−	4.25 (3.05-5.84)	<.001
* ERBB2*+/HR+ vs HR+/*ERBB2*−	1.94 (1.43-2.62)	<.001
* ERBB2*+/HR− vs HR+/*ERBB2*−	2.81 (2.02-3.85)	<.001
Unknown vs HR+/*ERBB2*−	1.34 (1.05-1.73)	.02
Age, ≤60 years vs >60 years	1.64 (1.38-1.96)	<.001
Rural residence, yes vs no	1.24 (0.96-1.59)	.09
Income quintile		
2 vs 1	1.07 (0.82-1.40)	.61
3 vs 1	1.07 (0.81-1.40)	.64
4 vs 1	0.94 (0.71-1.24)	.65
5 vs 1	1.08 (0.82-1.42)	.59
VW Elixhauser comorbidity index		
Score <0 vs 0	0.95 (0.73-1.21)	.67
Score 1-4 vs 0	1.03 (0.84-1.26)	.80
Score ≥5 vs 0	0.94 (0.72-1.21)	.65
Index year at diagnosis^b^	0.94 (0.90-0.98)	.01

Abbreviations: HR, hormone receptor; TNBC, triple-negative breast cancer.

^a^ HR+/*ERBB2*− was treated as the referent group, as it had the lowest hazard value.

^b^ Continuous from 2009 to 2017.

### Characteristics of Patients Treated With Radiotherapy for Brain Metastases

The median (IQR) age of patients treated with radiotherapy for breast cancer BRM was 56 (48-65) years (eTable 5 in the Supplement); those treated with SRS were slightly younger than those treated with WBRT (51 years vs 58 years, *P* < .001). Most patients lived in an urban setting (479 [87.2%]) as opposed to a rural setting. Similarly, likelihood of treatment with SRS vs WBRT did not differ by patients’ income quintiles nor the VW Elixhauser comorbidity index. The percentage of patients treated with SRS, as opposed to WBRT, by year of MBC diagnosis was 11.6% among patients diagnosed from 2009 to 2010, 13.5% from 2011 to 2012, 17.6% from 2013 to 2014, and 31.4% from 2015 to 2016 (*P* < .001).

The median survival time from first radiotherapy for BRM was 5.1 months in the overall cohort and 4.8 months for HR-positive/*ERBB2-*negative cancer, 8.7 months for *ERBB2-*positive/HR-positive cancer, 9.4 months for *ERBB2-*positive/HR-negative cancer, 2.6 months for TNBC, and 3.9 months for the unknown subtype cohort (Table 2). A univariable and multivariable Cox model for overall survival among women with breast cancer brain metastases (n = 549) is outlined in eTable 6 in the Supplement.

## Discussion

To our knowledge, this is the first population-based study illustrating cumulative risk of BRM requiring radiotherapy among patients with MBC. We found that overall, 14% (approximately 1 in 7) women in Ontario diagnosed with de novo MBC between 2009 and 2018 received radiotherapy for BRM after a median follow-up of 19.3 months.

Our findings confirm that patients with *ERBB2-*positive and triple-negative MBC are at highest risk of requiring radiotherapy for BRM. In a Surveillance, Epidemiology, and End Results study that reported the prevalence of BRM at the time of initial diagnosis of MBC (but did not capture the subsequent incidence of BRM), BRM were also most common in patients with *ERBB2-*positive /HR-negative (11.5%) and triple-negative (11.4%) subtypes^19^ The significantly higher prevalence of BRM at initial diagnosis in that study compared to our study may be because of more frequent routine imaging of the brain in the US or a more comprehensive database that captures BRM irrespective of treatment with radiotherapy. In the French Epidemiological Strategy and Medical Economics database, 16 701 patients diagnosed with MBC between January 2008 and December 2014 were tracked longitudinally.^20^ BRM were diagnosed in 24.6% of patients after a median follow-up of 42.8 months.^20^ However, detailed data regarding time to development of BRM by BC subtype was not reported. In the Systemic Therapies for HER2-positive Metastatic Breast Cancer study of 977 patients with *ERBB2-*positive MBC, 87 (8.9%) had evidence of BRM at initial diagnosis of metastatic disease while 212 (21.7%) subsequently developed intracranial metastatic disease, but any difference in incidence over time was not recorded.^21^ BRM incidence rates for each subtype are similarly lacking in the Breast Cancer Network Registry study of 1712 MBC patients with BRM,^22^ but detailed data regarding their survival patterns were well documented.

This study contributed novel data to the literature by highlighting the time to treatment of BRM from diagnosis of MBC for each subtype.^23,24,25^ This was 15.4 months across the entire cohort and varied from a median of 7.5 months for TNBC to a median of 19.8 months for *ERBB2-*positive/HR-positive MBC. Observed differences in the time to development of BRM by breast cancer subtype stimulate important questions about the mechanism of intracranial metastatic spread. While certain breast cancer subtypes may have an intrinsic propensity to metastasize to the brain, it is also possible that shorter time to development of BRM may simply be related to tumor growth rate. A better understanding of subtype specific BRM biology requires further study and may ultimately help identify potential preventative interventions.

Our study also provides detailed data regarding the risk of death over time among patients with MBC by breast cancer subtype. The annual risk of death was lowest among patients with *ERBB2-*positive MBC in our study. While *ERBB2 *targeting medications were historically thought to have low central nervous system (CNS) activity due to their inability to cross the blood-brain barrier, there is growing evidence that BRM as well as brain radiation can disrupt this membrane, which increases the permeability of systemic therapies into the CNS.^26,27,28,29^ Dijkers et al^30^ have shown that even bulky *ERBB2* antibodies have a degree of CNS penetration in radiolabeled positron emission tomography studies. This is further supported by the recent KAMILLA trial, which demonstrates that T-DM1 is effective among patients with *ERBB2-*positive breast cancer BRM.^31^ Hence, it is not surprising that in our cohort patients with *ERBB2-*positive breast cancer BRM had the lowest cumulative risk of death and longest OS after treatment with brain radiotherapy. With recent data demonstrating intracranial efficacy of small molecule tyrosine-kinase inhibitors (particularly tucatinib) in *ERBB2-*positive MBC,^28,32^ the survival of patients with *ERBB2-*positive breast cancer BRM is likely to improve even further over time.

Surprisingly, despite a generally favorable prognosis of women with metastatic HR-positive/*ERBB2-*negative breast cancer with median survival now exceeding 5 years,^33^ survival among that subgroup in our study was relatively short. Most patients did not have access to CKD4/6 inhibitors during the time period of our study. Hence, the survival benefits associated with these drugs, which are now first line standard-of-care for patients with HR-positive/*ERBB2-*negative MBC, are not reflected in this study.^34,35,36,37^ On the other hand whether CDK4/6 inhibitors have any impact on the course of BRM is unclear. Survival was shortest among patients with TNBC, with the majority of patients (60.5%) dying within 12 months of diagnosis. The lack of targeted systemic therapies for patients with TNBC leads to generally poor control of extracranial metastases and short survival. Lin et al^38^ demonstrated that death from isolated BRM is uncommon among women with metastatic TNBC and generally occurs in the context of systemic disease progression; hence, extracranial disease is felt to dominate prognosis in this patient population.

We found that median survival from the time of radiotherapy for breast cancer BRM was significantly longer among patients treated with SRS compared with WBRT, even after adjustment for breast cancer subtype, patient age, residence setting (urban vs rural), weighted VW Elixhauser comorbidity index, income status, and index year at diagnosis. Although patients who undergo SRS have better outcomes, this may be due to selection bias rather than treatment efficacy since SRS is typically offered to patients who are healthier and have a lower burden of intracranial metastatic disease.

Our data have important implications for potential future BRM screening programs (Table 4). In light of a high cumulative incidence of BRM and comparatively low risk of death, patients with *ERBB2-*positive MBC may be ideal candidates for BRM screening, particularly in the setting of systemic therapies with known CNS efficacy^31,32,39,40,41^ and survival benefits that extend to patients with active BRM.^42^ Further, given an almost linear increase in incidence of BRM for at least 5 years after a MBC diagnosis, ongoing long-term screening for intracranial metastatic disease may be indicated among patients with *ERBB2-*positive MBC. In contrast, the low annual incidence of BRM among patients with HR-positive/*ERBB2-*negative breast cancer would not justify screening for intracranial metastatic disease in this patient population. Finally, although patients with metastatic TNBC have a high cumulative incidence of BRM, it is unclear whether these patients would benefit from early detection and treatment of intracranial metastatic disease given their typically aggressive disease biology. However, the aggressiveness of TNBC varies considerably; some patients with indolent disease biology or disease that is well controlled on systemic therapy could be considered for screening, ideally in a research setting.

**Table 4.** Suggested Recommendations for Brain Metastases Screening Among Patients With Metastatic Breast Cancer, Based on Population-Based Estimates of Cumulative Incidence of Intracranial Metastatic Disease

Variables	Patient, No. (%)			
	*ERBB2*+/HR+ (n = 310)	*ERBB2*+/HR− (n = 200)	TNBC (n = 258)	HR+/*ERBB2*− (n = 1215)
Proportion of patients alive from 0-24+ months since MBC diagnosis				
≥4 wk	303 (97.7)	192 (96.0)	238 (92.2)	1194 (98.3)
≥12 mo	235 (75.8)	140 (70.0)	102 (39.5)	953 (78.4)
≥24 mo	168 (54.2)	97 (48.5)	48 (18.6)	657 (54.1)
Incidence of surviving patients treated with radiotherapy from 0-24+ months since diagnosis with MBC				
BRM treated				
≤4 weeks of MBC diagnosis	6 (1.9)	1-5 (up to 2.5)^a^	1-5 (up to 1.9)^a^	9 (0.7)
≤12 mo of MBC diagnosis	16 (5.3)	22 (11.5)	32 (13.4)	46 (3.9)
>12 mo to ≤24 mo from MBC diagnosis	25 (10.6)	13 (9.3)	15 (14.7)	33 (3.5)
>24 mo from MBC diagnosis	23 (13.7)	19 (19.6)	7 (14.6)	44 (6.7)
Incidence per 100 person-years				
No. of BRM	64	54	54	123
Total person-years	703.9	401.1	272.4	2874.2
Incidence (per 100 person-years)	9.1	13.5	19.8	4.3
Proposed parameters for future BRM screening programs				
Possible use for BRM screening	Yes	Yes	Uncertain	No
Duration of screening^b^	Life-long	Life-long	Uncertain	NA
Frequency of screening^b^	Every 6 mo	Every 6 mo	Every 6 mo	NA

Abbreviations: BRM, brain metastases; HR, hormone receptor; MBC, metastatic breast cancer; NA, not applicable; TNBC, triple-negative breast cancer.

^a^ Data suppressed because of small numbers.

^b^ Data that may inform an optimal duration and frequency of screening is not yet available. Such data may emerge as part of ongoing prospective clinical trials.

Current American Society of Clinical Oncology and National Comprehensive Cancer Network guidelines do not support screening the brain of MBC patients for BRM given lack of supportive data. However, in an era of minimally toxic and precise stereotactic radiosurgery (SRS) as well as novel systemic therapies with intracranial efficacy, the potential utility of a BRM screening program is being reevaluated. In fact, the 2021 European Association of Neuro-Oncology and European Society for Medical Oncology guidelines indicate that screening at diagnosis is potentially justified in patients with metastatic *ERBB2-*positive and TNBC. The possible role of BRM screening among patients with MBC is the subject of a number of ongoing prospective trials (NCT03881605, NCT04030507, NCT03617341), some of which assess important patient-reported outcomes, toxicity measures, and other factors that were not captured in our study.

### Limitations

This study had limitations. The breast cancer subtype was unknown for a significant proportion (49.4%) of patients who developed BRM, which limited subtype-specific analyses. We also limited our study to patients with de novo MBC given that data regarding metastatic recurrence among patients with stage I to stage III breast cancer is lacking in the ICES database; this limits generalizability of our results because the natural history of de novo and recurrent MBC may differ. Furthermore, while our retrospective population-based study design allowed us to explore outcomes of all patients with BRM who were treated with radiotherapy in Ontario, the use of population level data are limited in its detail regarding the burden of intracranial disease, type of intracranial disease involvement (parenchymal BRM vs leptomeningeal disease), systemic therapy, presence vs absence of surgical interventions, as well as the cause of death. Given the constraints of population-based databases, we were also unable to explore granular data regarding treatment toxicity, such as neurologic symptoms and cognitive outcomes. Finally, it is acknowledged that a diagnosis of BRM was inferred by treatment with radiotherapy, since a specific diagnostic code for BRM does not exist in the ICES database. However, this is unlikely to be a significant underestimation as a recently published Ontario study demonstrated that an overwhelming majority (approximately 91%) of breast cancer patients with BRM received radiotherapy.^43^

## Conclusions

The findings of this study suggest that approximately 1 in 7 patients diagnosed with MBC will require radiotherapy for BRM; this proportion is even higher among patients with *ERBB2-*positive and triple-negative MBC, potentially justifying screening for intracranial metastatic disease in these patient populations. Marked differences in the incidence of BRM and time to development of BRM based on breast cancer subtype highlight intrinsic differences in the disease biology and propensity of breast cancer to metastasize to the brain. Further investigation of biologic mechanisms that result in intracranial metastatic spread may help identify potential preventative strategies.

## Notes

Supplement. **eTable 1.** List and Description of Datasets Available to ICES **eTable 2.** Inclusion and Exclusion Criteria **eTable 3.** Baseline Characteristics of Patients With Metastatic Breast Cancer Who Did vs Did Not Develop Brain Metastases **eTable 4.** Cumulative Incidence of Brain Metastases and Cumulative Incidence of Death in the Overall Patient Population **eTable 5.** Baseline Characteristics of Patients with Breast Cancer Brain Metastases Undergoing Radiation Therapy **eTable 6.** Univariable and Multivariable Cox Model for Overall Survival Among Women With Breast Cancer Brain Metastases

## References

[1] Tabouret E, Chinot O, Metellus P, Tallet A, Viens P, Gonçalves A. Recent trends in epidemiology of brain metastases: an overview. Anticancer Res. 2012;32(11):4655-4662. 23155227

[2] Witzel I, Oliveira-Ferrer L, Pantel K, Müller V, Wikman H. Breast cancer brain metastases: biology and new clinical perspectives. Breast Cancer Res. 2016;18(1):8. doi:10.1186/s13058-015-0665-1 26781299 PMC4717619

[3] Niikura N, Saji S, Tokuda Y, Iwata H. Brain metastases in breast cancer. Jpn J Clin Oncol. 2014;44(12):1133-1140. doi:10.1093/jjco/hyu156 25320339

[4] Jin J, Gao Y, Zhang J, . Incidence, pattern and prognosis of brain metastases in patients with metastatic triple-negative breast cancer. BMC Cancer. 2018;18(1):446. doi:10.1186/s12885-018-4371-0 29673325 PMC5909254

[5] Frisk G, Svensson T, Bäcklund LM, Lidbrink E, Blomqvist P, Smedby KE. Incidence and time trends of brain metastases admissions among breast cancer patients in Sweden. Br J Cancer. 2012;106(11):1850-1853. doi:10.1038/bjc.2012.163 22531629 PMC3364124

[6] Vaz-Luis I, Ottesen RA, Hughes ME, . Impact of hormone receptor status on patterns of recurrence and clinical outcomes among patients with human epidermal growth factor-2-positive breast cancer in the National Comprehensive Cancer Network: a prospective cohort study. Breast Cancer Res. 2012;14(5):R129. doi:10.1186/bcr3324 23025714 PMC4053106

[7] Kim YJ, Kim JS, Kim IA. Molecular subtype predicts incidence and prognosis of brain metastasis from breast cancer in SEER database. J Cancer Res Clin Oncol. 2018;144(9):1803-1816. doi:10.1007/s00432-018-2697-2 29971531 PMC11813522

[8] Lin NU, Vanderplas A, Hughes ME, . Clinicopathologic features, patterns of recurrence, and survival among women with triple-negative breast cancer in the National Comprehensive Cancer Network. Cancer. 2012;118(22):5463-5472. doi:10.1002/cncr.27581 22544643 PMC3611659

[9] Kuksis M, Gao Y, Tran W, . The incidence of brain metastases among patients with metastatic breast cancer: a systematic review and meta-analysis. Neuro Oncol. 2021;23(6):894-904. doi:10.1093/neuonc/noaa285 33367836 PMC8168821

[10] Komorowski AS, Warner E, MacKay HJ, Sahgal A, Pritchard KI, Jerzak KJ. Incidence of brain metastases in nonmetastatic and metastatic breast cancer: is there a role for screening? Clin Breast Cancer. 2020;20(1):e54-e64. doi:10.1016/j.clbc.2019.06.007 31447286

[11] Arnaout A, Varela NP, Allarakhia M, . Baseline staging imaging for distant metastasis in women with stages I, II, and III breast cancer. Curr Oncol. 2020;27(2):e123-e145. doi:10.3747/co.27.6147 32489262 PMC7253735

[12] Ramakrishna N, Anders CK, Lin NU, . Management of Advanced Human Epidermal Growth Factor Receptor 2-Positive Breast Cancer and Brain Metastases: ASCO Guideline Update. J Clin Oncol. 2022;JCO2200520:JCO2200520. doi:10.1200/JCO.22.00520 35947812

[13] Cardoso F, Senkus E, Costa A, . 4th ESO–ESMO international consensus guidelines for advanced breast cancer (ABC 4). Ann Oncol. 2018;29(8):1634-1657. doi:10.1093/annonc/mdy192 30032243 PMC7360146

[14] Giordano SH, Elias AD, Gradishar WJ. NCCN guidelines updates: Breast cancer. J Natl Compr Canc Netw. 2018;16(5S):605-610. doi:10.6004/jnccn.2018.0043 29784737

[15] Munro A, Alasia A, Bollman RD. *Self-Contained Labour Areas: A Proposed Delineation and Classification by Degree of Rurality. Data and Definitions*. Statistics Canada. 2011;8(2008008).

[16] van Walraven C, Austin PC, Jennings A, Quan H, Forster AJ. A modification of the Elixhauser comorbidity measures into a point system for hospital death using administrative data. Med Care. 2009;47(6):626-633. doi:10.1097/MLR.0b013e31819432e5 19433995

[17] Government of Canada SC. Health System Indicators (Canadian Institute for Health Information – CIHI). Statistics Canada; 2013.

[18] Collaborative Staging Task Force of the American Joint Committee on Cancer. Collaborative staging manual and coding instructions, version 1.0. American Joint Committee on Cancer and U.S. Department of Health and Human Services. Published 2004. Accessed July 8, 2022. https://corpora.tika.apache.org/base/docs/govdocs1/085/085260.pdf

[19] Martin AM, Cagney DN, Catalano PJ, . Brain metastases in newly diagnosed breast cancer: a population-based study. JAMA Oncol. 2017;3(8):1069-1077. doi:10.1001/jamaoncol.2017.0001 28301662 PMC5824221

[20] Pasquier D, Darlix A, Louvel G, . Treatment and outcomes in patients with central nervous system metastases from breast cancer in the real-life ESME MBC cohort. Eur J Cancer. 2020;125:22-30. doi:10.1016/j.ejca.2019.11.001 31835235

[21] Hurvitz SA, O’Shaughnessy J, Mason G, . Central nervous system metastasis in patients with her2-positive metastatic breast cancer: patient characteristics, treatment, and survival from SystHERs. Clin Cancer Res. 2019;25(8):2433-2441. doi:10.1158/1078-0432.CCR-18-2366 30593513

[22] Witzel I, Laakmann E, Weide R, . Treatment and outcomes of patients in the Brain Metastases in Breast Cancer Network Registry. Eur J Cancer. 2018;102:1-9. doi:10.1016/j.ejca.2018.07.004 30099223

[23] DiStefano A, Yong Yap Y, Hortobagyi GN, Blumenschein GR. The natural history of breast cancer patients with brain metastases. Cancer. 1979;44(5):1913-1918. doi:10.1002/1097-0142(197911)44:5<1913::AID-CNCR2820440554>3.0.CO;2-D 498057

[24] Chang EL, Lo S. Diagnosis and management of central nervous system metastases from breast cancer. Oncologist. 2003;8(5):398-410. doi:10.1634/theoncologist.8-5-398 14530493

[25] Lee SS, Ahn JH, Kim MK, . Brain metastases in breast cancer: prognostic factors and management. Breast Cancer Res Treat. 2008;111(3):523-530. doi:10.1007/s10549-007-9806-2 17990100

[26] Olson EM, Najita JS, Sohl J, . Clinical outcomes and treatment practice patterns of patients with HER2-positive metastatic breast cancer in the post-trastuzumab era. Breast. 2013;22(4):525-531. doi:10.1016/j.breast.2012.12.006 23352568 PMC3713786

[27] Vaz-Luis I, Ottesen RA, Hughes ME, . Impact of hormone receptor status on patterns of recurrence and clinical outcomes among patients with human epidermal growth factor-2-positive breast cancer in the National Comprehensive Cancer Network: a prospective cohort study. Breast Cancer Res. 2012;14(5):R129. doi:10.1186/bcr3324 23025714 PMC4053106

[28] Bachelot T, Romieu G, Campone M, . Lapatinib plus capecitabine in patients with previously untreated brain metastases from HER2-positive metastatic breast cancer (LANDSCAPE): a single-group phase 2 study. Lancet Oncol. 2013;14(1):64-71. doi:10.1016/S1470-2045(12)70432-1 23122784

[29] Lockman PR, Mittapalli RK, Taskar KS, . Heterogeneous blood-tumor barrier permeability determines drug efficacy in experimental brain metastases of breast cancer. Clin Cancer Res. 2010;16(23):5664-5678. doi:10.1158/1078-0432.CCR-10-1564 20829328 PMC2999649

[30] Dijkers EC, Oude Munnink TH, Kosterink JG, . Biodistribution of 89Zr-trastuzumab and PET imaging of HER2-positive lesions in patients with metastatic breast cancer. Clin Pharmacol Ther. 2010;87(5):586-592. doi:10.1038/clpt.2010.12 20357763

[31] Montemurro F, Delaloge S, Barrios CH, . Trastuzumab emtansine (T-DM1) in patients with HER2-positive metastatic breast cancer and brain metastases: exploratory final analysis of cohort 1 from KAMILLA, a single-arm phase IIIb clinical trial. Ann Oncol. 2020;31(10):1350-1358. doi:10.1016/j.annonc.2020.06.020 32634611

[32] Murthy RK, Loi S, Okines A, . Tucatinib, trastuzumab, and capecitabine for HER2-positive metastatic breast cancer. N Engl J Med. 2020;382(7):597-609. doi:10.1056/NEJMoa1914609 31825569

[33] Hortobagyi GN, Stemmer SM, Burris HA III, . Overall survival results from the phase III MONALEESA-2 trial of postmenopausal patients with hormone receptor–positive/human epidermal growth factor receptor 2–negative advanced breast cancer treated with endocrine therapy ± ribociclib. ESMO Congress 2021. Presented September 19, 2021.

[34] Slamon DJ, Neven P, Chia S, . Overall survival with ribociclib plus fulvestrant in advanced breast cancer. N Engl J Med. 2020;382(6):514-524. doi:10.1056/NEJMoa1911149 31826360

[35] Slamon DJ, Neven P, Chia S, . Ribociclib plus fulvestrant for postmenopausal women with hormone receptor-positive, human epidermal growth factor receptor 2-negative advanced breast cancer in the phase III randomized MONALEESA-3 trial: updated overall survival. Ann Oncol. 2021;32(8):1015-1024. doi:10.1016/j.annonc.2021.05.353 34102253

[36] Im SA, Lu YS, Bardia A, . Overall survival with ribociclib plus endocrine therapy in breast cancer. N Engl J Med. 2019;381(4):307-316. doi:10.1056/NEJMoa1903765 31166679

[37] Sledge GW Jr, Toi M, Neven P, . The effect of abemaciclib plus fulvestrant on overall survival in hormone receptor-positive, *ERBB2*-negative breast cancer that progressed on endocrine therapy-MONARCH 2: a randomized clinical trial. JAMA Oncol. 2020;6(1):116-124. doi:10.1001/jamaoncol.2019.4782 31563959 PMC6777264

[38] Lin NU, Claus E, Sohl J, Razzak AR, Arnaout A, Winer EP. Sites of distant recurrence and clinical outcomes in patients with metastatic triple-negative breast cancer: high incidence of central nervous system metastases. Cancer. 2008;113(10):2638-2645. doi:10.1002/cncr.23930 18833576 PMC2835546

[39] Soffietti R, Ahluwalia M, Lin N, Rudà R. Management of brain metastases according to molecular subtypes. Nat Rev Neurol. 2020;16(10):557-574. doi:10.1038/s41582-020-0391-x 32873927

[40] Hamilton EP, Shapiro CL, Boni V, . Primary analysis from DS8201-A-U105: A 2-part, open label, phase 1b trial assessing trastuzumab deruxtecan (T-DXd) with nivolumab (nivo) in patients (pts) with HER2-expressing advanced breast cancer. Ann Oncol. 2022;33(suppl 3):S194-S223. doi:10.1016/j.annonc.2022.03.181

[41] Pérez-García JM, Batista MV, Cortez P, . Trastuzumab Deruxtecan in Patients with Central Nervous System Involvement from HER2-Positive Breast Cancer: The DEBBRAH Trial. Neuro Oncol. 2022;•••:noac144; Epub ahead of print. doi:10.1093/neuonc/noac144 35639825 PMC9825345

[42] Lin NU, Borges V, Anders C, . Intracranial efficacy and survival with tucatinib plus trastuzumab and capecitabine for previously treated HER2-positive breast cancer with brain metastases in the HER2CLIMB trial. J Clin Oncol. 2020;38(23):2610-2619. doi:10.1200/JCO.20.00775 32468955 PMC7403000

[43] Gao YK, Kuksis M, Id Said B, . Treatment patterns and outcomes of women with symptomatic and asymptomatic breast cancer brain metastases: a single-center retrospective study. Oncologist. 2021;26(11):e1951-e1961. doi:10.1002/onco.13965 34506676 PMC8571756

