# Supplementary material for: Analysis of Rates of Brain Metastases and Association With Breast Cancer Subtypes in Ontario, Canada
Source: JAMA Netw Open. 2022 Aug 12;5(8):e2225424. doi: 10.1001/jamanetworkopen.2022.25424 (PMC9375172; doi:10.1001/jamanetworkopen.2022.25424)
Supplement: Supplement. — eTable 1. List and Description of Datasets Available to ICES eTable 2. Inclusion and Exclusion Criteria eTable 3. Baseline Characteristics of Patients With Metastatic Breast Cancer Who Did vs Did Not Develop Brain Metastases eTable 4. Cumulative Incidence of Brain Metastases and Cumulative Incidence of Death in the Overall Patient Population eTable 5. Baseline Characteristics of Patients with Breast Cancer Brain Metastases Undergoing Radiation Therapy eTable 6. Univariable and Multivariable Cox Model for Overall Survival Among Women With Breast Cancer Brain Metastases [file jamanetwopen-e2225424-s001.pdf]

Supplemental Online Content

Wang XY, Rosen MN, Chehade R, et al. Analysis of rates of brain metastases and association with breast cancer subtypes in Ontario, Canada. *JAMA Netw Open*. 2022;5(8):e2225424. doi:10.1001/jamanetworkopen.2022.25424

eTable 1. List and Description of Datasets Available to ICES
eTable 2. Inclusion and Exclusion Criteria
eTable 3. Baseline Characteristics of Patients With Metastatic Breast Cancer Who Did vs Did Not Develop Brain Metastases
eTable 4. Cumulative Incidence of Brain Metastases and Cumulative Incidence of Death in the Overall Patient Population
eTable 5. Baseline Characteristics of Patients with Breast Cancer Brain Metastases Undergoing Radiation Therapy
eTable 6. Univariable and Multivariable Cox Model for Overall Survival Among Women With Breast Cancer Brain Metastases

This supplemental material has been provided by the authors to give readers additional information about their work.

© 2022 Wang XY et al. *JAMA Network Open.*

eTable 1. List and Description of Datasets Available to ICES

Dataset	Related Variable	Description
**Owner: Cancer Care Ontario**		
Cancer Activity Level Reporting (ALR) *2005*	- Systemic therapy<br>- Supportive drugs<br>- Radiation therapy	Contains patient level activity within the cancer system focused on radiation and systemic therapy services and outpatient oncology clinic visits.
**Owner: Ontario Ministry of Health and Long term Care**		
Client Agency Program Enrolment (CAPE) *1999*	- Capitation costs	Registry of patients enrolled in a primary care model. Data elements include program type (family health team, family health organization, family health network, etc.) and patient enrolment status.
Home Care Database (HCD), Ontario Home Care Administrative System (OHCAS) *1990*	- Home care services	Captures information on all services provided or coordinated by Ontario Community Care Access Centres, including client data, intake and assessment information, admission and discharge, etc.
New Drug Funding Program (NDFP) *1995*	- Medication use including systemic therapy	Administered by Cancer Care Ontario, the NDFP funds new, and often very expensive, cancer drugs.
Ontario Cancer Registry (OCR) *1964*	- Cancer diagnosis<br>- Tumour characteristics (molecular)	Contains the diagnosis code for invasive cancer (International Classification of Diseases version 10) and the date of diagnosis for all residents of Ontario.
Ontario Drug Benefit (ODB) claims *1990*	- Medication use including systemic therapy	Contains claims for oral prescription drugs covered under the ODB program. Primarily includes drug claims for individuals 65 years of age and older, but also coverage under special ODB programs.
Ontario Health Insurance Plan (OHIP) claims database *1991*	- Outpatient physician visits<br>- Laboratory services<br>- Non-physician services<br>- Physician services (including costs related to breast reconstruction)	Contains claims paid by OHIP, the universal, single-payer provincial health insurance plan, for services provided by all eligible health care providers, including physicians (primary and specialist), groups, and laboratories.
Registered Persons Database (RPDB) *1991*	- Health service subscriber data	Contains birth and death dates, age, sex, and date of last contact with health care services in Ontario.
**Owner: Canadian Institute for Health Information**		
Continuing Care Reporting System (CCRS) *1996*	- Complex continuing care (CCC)<br>- Long-term care (LTC)	Contains information about residents receiving facility-based continuing care services. Range of services includes CCC, extended or chronic care, and residential care providing nursing services (that is, long-term care).
Discharge Abstract Database (DAD) *1988*	- Inpatient hospitalizations	Contains demographic, clinical, and administrative data for inpatient hospital admissions (patient separations).
National Ambulatory Care Reporting System (NACRS) *2000/2003*	- Ambulatory emergency department visit, dialysis clinic visits, and cancer clinic visits	Contains data from hospital- and community-based ambulatory care services, including same day surgery, outpatient clinics, and emergency departments.

© 2022 Wang XY et al. *JAMA Network Open.*

Dataset	Related Variable	Description
**Owner: Canadian Institute for Health Information**		
National Rehabilitation Reporting System (NRS) *2000*	- Rehabilitation admissions	Contains client data from adult inpatient rehabilitation facilities, such as administrative data (referral, admission, and discharge) and health and functional characteristics.
Ontario Mental Health Reporting System (OMHRS) *2005*	- Mental health admissions	Contains data on patients in adult designated inpatient mental health beds in acute and psychiatric facilities. Data elements include admission and discharge dates, diagnoses, service utilization, etc.

Reference: ICES Data Dictionary [Internet]. 2019. Available from: https://datadictionary.ices.on.ca/Applications/DataDictionary/Default.aspx

© 2022 Wang XY et al. *JAMA Network Open.*

eTable 2. Inclusion and Exclusion Criteria

	**Patients excluded**	**Patients remaining**
Cohort of Ontario women diagnosed with breast cancer between 01/01/2009 and 31/12/2018		100,747
**Exclusions**		
Previous cancer cases	13,332	87,415
Subsequent non-breast cancer cases	4,623	82,792
Age <18	583	82,209
Death before or on index date	448	81,761
Non-Ontario residents at index date	43	81,718
Ineligible for OHIP at index date	483	81,235
Patients with Stage 1-3 disease or unknown stage	77,319	3,916

© 2022 Wang XY et al. *JAMA Network Open.*

eTable 3. Baseline Characteristics of Patients With Metastatic Breast Cancer Who Did vs Did Not Develop Brain Metastases

(n=3,916)

Variable	No BRM (N=3,367)	BRM (N=549)	All (N=3,916)	p-value
**Breast Cancer Subtype, n (%)**				
TNBC	204 (79.1)	54 (20.9)	258 (6.6)	<.001
ERBB2+/HR+	246 (79.4)	64 (20.6)	310 (7.9)	
ERBB2+/HR-	146 (73.0)	54 (27.0)	200 (5.1)	
HR+/ERBB2-	1,092 (89.9)	123 (10.1)	1,215 (31.0)	
Unknown	1,679 (86.9)	254 (13.1)	1,933 (49.4)	
**Age at Treatment**				
Median (IQR)	64 (53-77)	56 (48-65)	63 (52-75)	<.001
≤60, n (%)	1,369 (40.7)	345 (62.8)	1,714 (43.8)	<.001
>60, n (%)	1,998 (59.3)	204 (37.2)	2,202 (56.2)	
**Location, n (%)**				
Urban	3,015 (89.5)	479 (87.2)	3,494 (89.2)	0.11
Rural	352 (10.5)	70 (12.8)	422 (10.8)	
**Income quintile, n (%)**				
1	733 (21.8)	100 (18.2)	833 (21.3)	0.38
2	771 (22.9)	127 (23.1)	898 (22.9)	
3	653 (19.4)	115 (20.9)	768 (19.6)	
4	624 (18.5)	102 (18.6)	726 (18.5)	
5	586 (17.4)	105 (19.1)	691 (17.6)	
**Elixhauser co-morbidity index, n (%)**				
Score < 0	461 (13.7)	83 (15.1)	544 (13.9)	<.001
Score = 0	1,311 (38.9)	231 (42.1)	1,542 (39.4)	
Score = 1-4	856 (25.4)	156 (28.4)	1,012 (25.8)	
Score ≥ 5	739 (21.9)	79 (14.4)	818 (20.9)	
**Index year at diagnosis, n (%)**				
2009-2010	614 (18.2)	138 (25.1)	752 (19.2)	<.001
2011-2012	619 (18.4)	133 (24.2)	752 (19.2)	
2013-2014	662 (19.7)	125 (22.8)	787 (20.1)	
2015-2016	713 (21.2)	102 (18.6)	815 (20.8)	
2017-2018	759 (22.5)	51 (9.3)	810 (20.7)	

Legend: BRM = brain metastases; HR= hormone receptor; MBC = metastatic breast cancer; TNBC= triple negative breast cancer.

© 2022 Wang XY et al. *JAMA Network Open.*

eTable 4. Cumulative Incidence of Brain Metastases and Cumulative Incidence of Death in the Overall Patient Population

Follow-up Time	Cumulative incidence of brain metastases (%, 95% CI)	Cumulative incidence of death (%, 95% CI)
**3 months**	3.1 (2.6–3.7)	14.4 (13.3–15.5)
**6 months**	4.0 (3.4–4.6)	21.0 (19.7–22.3)
**9 months**	4.9 (4.3–5.6)	25.8 (24.4-27.2)
**1 year**	6.2 (5.4–6.9)	29.6 (28.2–31.1)
**2 years**	10.1 (9.2–11.1)	42.3 (40.7-43.9)
**3 years**	12.6 (11.5–13.7)	52.4 (50.7–54.0)
**4 years**	14.5 (13.3–15.7)	59.0 (57.3–60.7)
**5 years**	15.5 (14.3–16.8)	64.4 (62.7–66.1)
**6 years**	16.4 (15.1–17.7)	68.8 (67.1–70.5)
**7 years**	16.6 (15.3–18.0)	71.6 (69.8–73.2)
**8 years**	16.7 (15.4-18.0)	73.7 (71.9–75.4)
**9 years**	16.9 (15.5–18.2)	75.9 (74.1–77.7)
**10 years**	N.R	76.3 (74.3–78.1)

Note: N.R, not reportable due to small sample size.

© 2022 Wang XY et al. *JAMA Network Open.*

eTable 5. Baseline Characteristics of Patients with Breast Cancer Brain Metastases Undergoing Radiation Therapy (n=549)

Variable	SRS (N=94)	WBRT (N=455)	All (N=549)	[a]p-value
**Breast Cancer Subtype, n (%)**				
TNBC	8 (8.5)	46 (10.1)	54 (9.8)	<.001
ERBB2+/HR+	23 (24.5)	41 (9.0)	64 (11.7)	
ERBB2+/HR-	14 (14.9)	40 (8.8)	54 (9.8)	
HR+/ERBB2-	16 (17.0)	107 (23.5)	123 (22.4)	
Unknown	33 (35.1)	221 (48.6)	254 (46.3)	
**Age at Treatment**				
Median (IQR)	51 (42-60)	58 (48-65)	56 (48-65)	<.001
≤60, n (%)	71 (75.5)	274 (60.2)	345 (62.8)	0.01
>61, n (%)	23 (24.5)	181 (39.8)	204 (37.2)	
**Location, n (%)**				
Urban	86 (91.5)	393 (86.4)	479 (87.2)	0.18
Rural	8 (8.5)	62 (13.6)	70 (12.8%)	
**Income quintile, n (%)**				
1	11 (11.7)	89 (19.6)	100 (18.2)	0.22
2	26 (27.7)	101 (22.2)	127 (23.1)	
3	16 (17.0)	99 (21.8)	115 (20.9)	
4	19 (20.2)	83 (18.2)	102 (18.6)	
5	22 (23.4)	83 (18.2)	105 (19.1)	
**Time from MBC diagnosis to BRM treatment (days)**				
Median (IQR)	630 (352-1,153)	435 (109-842)	461 (137-900)	<.001
**VW Elixhauser co-morbidity index, n (%)**				
Score < 0	18 (19.1)	65 (14.3)	83 (15.1)	0.37
Score = 0	39 (41.5)	192 (42.2)	231 (42.1)	
Score = 1-4	28 (29.8)	128 (28.1)	156 (28.4)	
Score ≥ 5	9 (9.6)	70 (15.4)	79 (14.4)	
**Index year at diagnosis, n (%)**				
2009-2010	16 (17.0)	122 (26.8)	138 (25.1)	<.001
2011-2012	18 (19.1)	115 (25.3)	133 (24.2)	
2013-2014	22 (23.4)	103 (22.6)	125 (22.8)	
2015-2016	32 (34.0)	70 (15.4)	102 (18.6)	

Legend: SRS = stereotactic radiosurgery, WBRT = whole brain radiotherapy, MBC = metastatic breast cancer, BRM = brain metastases

[a]p-value compares WBRT and SRS groups.

eTable 6. Univariable and Multivariable Cox Model for Overall Survival Among Women With Breast Cancer Brain Metastases

(n=549)

[a] Variable	Uni-variable Hazard Ratio (95% CI)	[b]p-value	Multi-variable Hazard Ratio (95% CI)	[b]p-value
**Treatment**				
SRS vs. WBRT	0.51 (0.39-0.66)	<.001	0.59 (0.45-0.77)	<.001
**Breast cancer subtype**				
TNBC vs. ERBB2+/HR-	2.72 (1.78-4.20)	<.001	2.49 (1.62-3.87)	<.001
ERBB2+/HR+ vs. ERBB2+/HR-	1.12 (0.73-1.74)	0.60	1.20 (0.78-1.88)	0.41
HR+/ERBB2- vs. ERBB2+/HR-	1.74 (1.20-2.58)	<.01	1.60 (1.10-2.37)	0.02
Unknown vs. ERBB2+/HR-	2.03 (1.45-2.93)	<.001	1.51 (1.05-2.25)	0.03
**Age**				
≥61 vs. <60	1.37 (1.13-1.65)	<.001	1.30 (1.07-1.58)	0.01
Rural residence				
Yes vs. no	0.96 (0.72-1.25)	0.76	0.88 (0.66-1.16)	0.38
**Income quintile**				
2 vs. 1	0.96 (0.73 - 1.27)	0.75	0.95 (0.71-1.26)	0.71
3 vs. 1	0.81 (0.61-1.09)	0.16	0.76 (0.56-1.01)	0.06
4 vs. 1	0.71 (0.53-0.97)	0.03	0.70 (0.52-0.95)	0.02
5 vs. 1	0.74 (0.55-1.00)	0.05	0.72 (0.53-0.98)	0.04
**Time from initial diagnosis of MBC to BRM treatment**				
Per 30-days	1.00 (1.00-1.01)	0.82	1.00 (1.00-1.01)	0.47
**VW Elixhauser co-morbidity index**				
Score <0 vs. 0	0.99 (0.75 - 1.29)	0.93	1.04 (0.79-1.36)	0.78
Score 1-4 vs. 0	1.02 (0.82-1.27)	0.86	1.14 (0.91-1.43)	0.25
Score >=5 vs. 0	0.97 (0.73 -1.28)	0.83	0.98 (0.73-1.29)	0.87
**Index year at diagnosis**				
Continuous from 2009 to 2018	0.91 (0.88-0.95)	<.0001	0.93 (0.88-0.97)	0.002

Legend: SRS = stereotactic radiosurgery, WBRT = whole brain radiotherapy, MBC = metastatic breast cancer, TNBC= triple negative breast cancer, HR= hormone receptor

[a]ERBB2+/HR- was treated as the reference group, as it had the lowest hazard value, which is different from the reference group for the outcome of time from MBC to BRM
[b]p-value compares WBRT and SRS groups.
